# Optimization of Delivery and Bioavailability of Encapsulated Caffeic Acid

**DOI:** 10.3390/foods12101993

**Published:** 2023-05-15

**Authors:** Monika Stanciauskaite, Monika Poskute, Vaida Kurapkiene, Mindaugas Marksa, Valdas Jakstas, Liudas Ivanauskas, Milda Kersiene, Daiva Leskauskaite, Kristina Ramanauskiene

**Affiliations:** 1 Institute of Pharmaceutical Technologies, Faculty of Pharmacy, Lithuanian University of Health Sciences, Sukileliai Avenue 13, LT-50162 Kaunas, Lithuania; 2 Department of Drug Chemistry, Faculty of Pharmacy, Lithuanian University of Health Sciences, Sukileliai Avenue 13, LT-50162 Kaunas, Lithuania; 3 Department of Clinical Pharmacy, Faculty of Pharmacy, Lithuanian University of Health Sciences, Sukileliai Avenue 13, LT-50162 Kaunas, Lithuania; 4 Department Analytical & Toxicological Chemistry, Faculty of Pharmacy, Lithuanian University of Health Sciences, Sukileliai Avenue 13, LT-50162 Kaunas, Lithuania; 5 Department of Food Science and Technology, Kaunas University of Technology, Radvilenu pl. 19, LT-50254 Kaunas, Lithuania

**Keywords:** antioxidant, caffeic acid, capsules, solubility

## Abstract

Caffeic acid is a widely distributed phenolic acid. It is described in the scientific literature that caffeic acid has poor solubility. The aim of this study was to improve the solubility of caffeic acid for better dissolution kinetics when administered orally. During the study, oral capsules of different compositions were modeled. The results of the disintegration test revealed that the excipients affected the disintegration time of the capsules. The excipient hypromellose prolonged the disintegration time and dissolution time of caffeic acid. The dissolution kinetics of caffeic acid from capsules depend on the chosen excipients. P407 was more effective compared to other excipients and positively affected the dissolution kinetics of caffeic acid compared to other excipients. When the capsule contained 25 mg of β-cyclodextrin, 85% of the caffeic acid was released after 60 min. When the capsule contained 25–50 mg poloxamer 407, more than 85.0% of the caffeic acid was released from capsules after 30 min. The research results showed that in order to improve the dissolution kinetics of caffeic acid, one of the important steps is to improve its solubility.

## 1. Introduction

In recent years, the food and food supplements industry has increasingly focused on plant-derived active compounds [1,2,3]. Various phenolics can be used as functional food additives. Phenolic acids have gained momentum due to their significant nutritional benefits and functions, such as antioxidant, anti-inflammatory, antimicrobial, cardioprotective, anticancer, antidiabetic properties, etc. [4]. One such phenolic acid, which is characterized by strong biological properties, is caffeic acid. Caffeic acid can be used as an antioxidant component in dietary supplements [5,6,7,8]. Caffeic acid is found naturally in various fruits, vegetables, or herbs, such as coffee, turmeric, apples, oats, rosemary, basil, wheat, olive oil, and narrow-leaved purple coneflower flowers [9,10]. Yang et al. in a direct kinase assay found that caffeic acid directly inhibits IRAK1 and IRAK4, thereby acting in an anti-inflammatory manner [11]. In a study, Wan et al. found that 500 mg/kg of chlorogenic acid can reduce colon inflammation. Intestinal microbiota in the colon can break down chlorogenic acid into caffeic acid, and for this reason, the authors suggest that caffeic acid may have a protective effect on colon inflammation [12]. However, most natural active compounds often have poor water solubility, with caffeic acid being no exception [8,13,14]. 

Currently, not only drugs but also daily components found in food supplements are being evaluated for their bioavailability absorption. Solubility and bioavailability are crucial parameters that determine the dose release of a substance. Scientific research is increasingly focusing on the bioavailability of components in food supplements, such as minerals and vitamins [15]. Research results show that caffeic acid has poor solubility and bioavailability [6,7,14]. It is known that for a drug to be effective it must be absorbed in the gastrointestinal tract [16]. CA is absorbed in the body by several mechanisms. The first mechanism of absorption is by passive diffusion in the stomach, as CA is not in an ionic form in acidic media [17,18,19]. A small fraction of CA is absorbed in the stomach via enterocytes in the wall of the gastrointestinal tract [18,19]. The second mechanism of CA absorption is by active transport through sodium ion channels in the small intestine [18]. Microbial esterase degrades the ester moiety of CA, and the free form of CA is absorbed in the intestinal mucosa [17]. 

Scientific reports suggest that 95% of CA is absorbed in the human small intestine [17,18], but studies in a rat intestinal epithelial model (Caco-2 cell monolayer) showed low CA cell permeation, with an absorption of 12.4% in the intestine of the rats [20]. In humans, both passive absorption in the stomach and active absorption in the small intestine can influence CA absorption [18]. In a study by Murad et al., caffeic acid inhibited HT-29 cell growth by modulating the cell cycle with increased apoptosis in human colon adenocarcinoma cells [21]. Ongoing research shows that studies on the bioabsorption of CA are relevant, and it is appropriate to investigate the potential for the application of caffeic acid in the modeling of oral forms. One of the possible solid forms is capsules, and it is therefore important to assess how the selected excipients will affect the dissolution kinetics of caffeic acid from capsules. 

The evaluation of the dissolution of the active ingredient is in relation to time and the influence of excipients on the dissolution kinetics [22,23]. It is important to select appropriate excipients. Excipients should be non-toxic, non-allergenic, and guarantee an effective therapeutic effect at the lowest therapeutic concentration of the active substance. In addition, they must be stable and comply with the requirements of the regulatory documents in order to give the desired consistency, durability, and stability to the dosage forms. Depending on the intended use, excipients are classified as dosage forms, stabilizers, prolongers, sensory modifiers (correctors), coloring agents, solubility enhancers, and bioavailability enhancers [16,17,18,19,20,21,22,23,24]. Microcrystalline cellulose, starch, and glucose are often used as fillers in capsule production. Microcrystalline cellulose is widely used as a filler in oral tablets and capsules [25,26,27]. 

Hypromellose is widely used in the manufacturing of solid oral dosage forms as a tablet binder, coating film, disintegration prevention agent for capsules, and as a substance prolonging drug release [26,28]. Cyclodextrins (CDs) are used in pharmaceuticals as fillers, dissolution enhancers, binders, and stabilizers [13,29,30]. Japanese researchers have found that β-cyclodextrins can improve the solubility of caffeic acid [8]. Poloxamers are non-ionic polyoxyethylene-polyoxypropylene copolymers mainly used in different formulations as emulsifiers or solvents [27,31,32]. Poloxamers swell and gel in water, thus prolonging the relaxation of the active substance [33]. There is evidence in the scientific literature on the solubility-enhancing properties of poloxamer materials, but the effect of poloxamer on the solubility of caffeic acid has not been investigated so far [32,33]. These excipients are used in the production of oral tablets, capsules, and liquid forms, which are widely used in modeling food supplements [34,35,36].

Advanced methods, such as nanoencapsulation and microencapsulation, are used to efficiently deliver phenolic acids. These techniques protect natural antioxidants from degradation, may allow controlled targeted release, and increase the availability and stability of active compounds [37,38]. The scientific literature contains sufficient research data on encapsulation techniques [39]. In the manufacturing of hard capsules, the mixture to be encapsulated is often in powder form, containing the active ingredient and excipients, some of which may be solubilizers. It is relevant to study the influence of excipients on the dissolution kinetics of caffeic acid. The aim of this work is to simulate capsules with different excipients and caffeic acid and to perform their dissolution evaluation in vitro. During this study, attention is paid to the influence of excipients on the dissolution kinetics of caffeic acid and its disintegration from the modeled hard capsules. 

## 2. Materials and Methods

### 2.1. Materials

All reagents, standards, and solvents were used as analytical grade. Caffeic acid (≥98%, HPLC) was purchased from Sigma-Aldrich Chemie GmBh (Steinheim, Germany). d(+)-glucose monohydrate, starch, hypromellose, poloxamer 407 (Kolliphor P 407), β-cyclodextrin, and PROSOLV SMCCTM 50 were obtained from Sigma-Aldrich GmbH (Buchs, Switzerland) and (Penwest, UK), respectively, and all were used as excipients. Ethanol (96%) was purchased from AB “Vilniaus degtine” (Vilnius, Lithuania). Phosphate-buffered saline (PBS) (pH~7.4) was obtained from Gibco (Paisley, UK). Ultrapure water was produced using a water purification system Milli-Q^®^ (Millipore, Arlington, MA, USA). Chromatographic grade acetonitrile and trifluoracetic acid (TFA) were obtained from Sigma-Aldrich Chemie GmbH (Steinheim, Germany).

### 2.2. In Vitro Gastrointestinal Digestion

Samples of capsules were digested using static in vitro simulation of the gastrointestinal food digestion model operated under adult conditions [40]. Digestion media were prepared according to Minekus et al. [41]. Briefly, 200 mg of caffeic acid with 2.00 g glass beads were mixed with 4 mL of distilled water and incubated at 37 °C with orbital motion 150 min^−1^ in a water bath (Thermolab, GFL 1092). After, this was mixed with 5 mL of simulated saliva containing amylase (75 U/mL) for 2 min and then mixed with 10 mL of simulated gastric juice containing pepsin (2000 U/mL of digesta). The gastric pH was corrected between 2 and 3 using 6 M HCl, and the final digest volume was adjusted up to 20 mL. For the analysis, samples were taken at different times of gastric digestion: 0 min, 15 min, 30 min, 60 min, and 120 min. Pepsin activity in the samples was stopped by the addition of NaOH until a pH of 7 was reached. All samples were cooled down to 0–4 °C in ice water and centrifugated at 4000 rpm at +4 °C temperature (MPW-260RH, MPW Med. Instruments, Warsaw, Poland). After centrifugation, the samples were filtered, and the soluble fraction was collected, frozen, and stored at −18 °C until analysis. Samples were lyophilized. The digestion procedure was performed in triplicate.

### 2.3. Solubility of Caffeic Acid 

The solubility of caffeic acid into the prepared solution of different excipients was evaluated. A fixed amount of caffeic acid (100 mg) was added into the tubes with different excipients and their mixtures (S1–S14), according to the compositions of capsules (Table 1). Purified water was used as a solvent. All samples were mixed by magnetic stirrer IKAMAG C-MAG HS7 (IKA-Werke GmbH & Co.KG, Staufen im Breisgau, Germany) at 1250 rpm for 30 min. After mixture, the tubes were placed into a shaking incubator (GFL, Germany) for 48 h at 40 °C temperature. An undissolved fraction of caffeic acid was separated using Eppendorf Centrifuge 5810R (Hamburg, Germany). The quantity of dissolved caffeic acid was determined by HPLC, in triplicate.

### 2.4. Encapsulation Process

Hard capsules in N0 size were used for production. The encapsulated mixture’s mixture form was powder. Powders for the capsules were prepared by the mixing of caffeic acid and each excipient. A capsule-filling machine (Capsuline, Davie, FL, USA) was used for the preparation of different compositions of capsules. The amount of caffeic acid was 100 mg in all samples. The amount of excipients was different and varied between 50 mg and 150 mg. The manufactured capsules were divided into four research groups: group I—used fillers d-(+)-glucose, PROSOLV SMCCTM 50 mixture, and starch, which should not modify the release of the active substance; group II—used hypromellose as a filler; in groups III and IV—excipients (poloxamer 407 and β-cyclodextrin) were used to improve the solubility of CA. The compositions of capsules are presented in Table 1.

### 2.5. Capsule Disintegration Test

The disintegration test of the capsules was performed according to Ph. Eur. 2.9.1 methodology. The disintegration time of the capsules was determined using the device IKAMAG C-MAG HS7 (IKA^®^-Werke GmbH & Co.KG, Staufen im Breisgau, Germany). Then, 0.1 M hydrochloric acid solution was chosen as the disintegration medium. The temperature of the medium was 37 ± 0.50 °C. The apparatus was operated for 30 min.

### 2.6. Capsule Dissolution Test

The dissolution test of the capsules was performed according to Ph. Eur. 2.9.3 methodology using the paddle apparatus Sotax AT 7smart (SOTAX AG, Allschwil, Switzerland). Distilled water was used as the dissolution medium. The volume of the medium was 500 mL at 37.0 ± 0.5 °C temperature. The rotation speed of the paddle was selected as 100 rpm. Samples from the dissolution medium were removed at time points of 5, 10, 15, 30, 45, and 60 min and replaced by the same volume of fresh dissolution medium. The analysis of the caffeic acid was performed by the HPLC method.

### 2.7. Qualitative and Quantitative Analyses of Caffeic Acid by HPLC Method

Caffeic acid was determined in all tested samples by validated high-performance liquid chromatography (HPLC). Qualitative and quantitative analyses were performed using the Waters 2695 Alliance system (Waters, Milford, MA, USA) equipped with a Waters 2998 photodiode array detector. Separation was performed using an ACE column (C18, 250 mm × 4.6 mm, particle size 5 µm). The mobile phase of the chromatographic method consisted of eluent A (0.1% TFA) and eluent B (acetonitrile). The gradient variation consisted of the following: 0–1 min—2% B, 1–20 min—2–98% B, 20–23 min—98% B, 23–24 min—98–2% B. The eluent flow rate was 1 mL/min and injection volume was 10 µL. The column was temperature-controlled and maintained at 30 °C. The determination of caffeic acid was performed at 320 nm wavelength.

### 2.8. Statistical Analysis

A statistical analysis of experimental data was performed using SPSS software (version 20.0) and Microsoft Office Excel 2019. All results were presented as the mean and standard deviation of three consecutive assay results. A one-way ANOVA with Tukey’s test was used for the statistical analysis. A statistically significant difference was determined at *p* < 0.05. 

## 3. Results

### 3.1. In Vitro Gastrointestinal Digestion of Caffeic Acid

**Figure 1:**
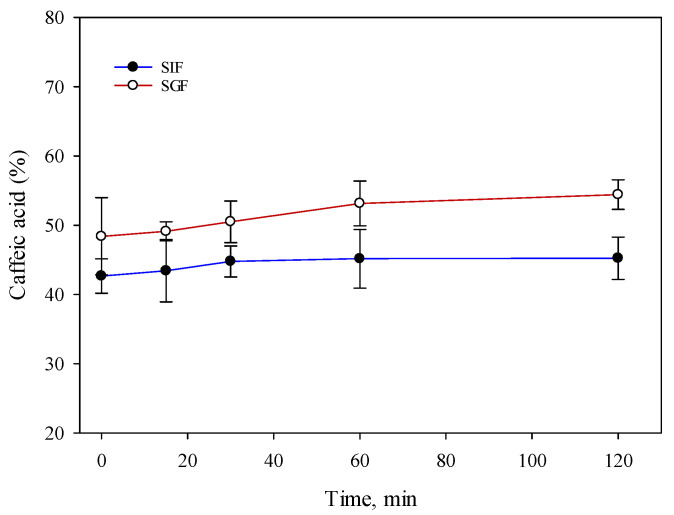
Solubility properties during in vitro digestion study of caffeic acid (SGF—simulated gastric fluid; SIF—simulated intestinal fluid) (mean, SD, n = 3).

In order to produce oral solid capsules’ formulations with caffeic acid, the solubility of caffeic acid in in vitro gastrointestinal digestion was investigated. In vitro gastrointestinal digestion is a commonly used method to simulate the process of digestion in the human body. This method is used to evaluate the performance of capsules, particularly those that are designed to release their contents in a specific location within the gastrointestinal tract. The results of the in vitro gastrointestinal digestion are shown in Figure 1.

The results showed that the higher amount of caffeic acid dissolved in the gastric media. A statistical analysis of the data showed a statistically significant (*p* < 0.05) difference between the dissolved content of caffeic acid in gastric fluid (54.4 ± 2.1%) compared to intestinal fluid (45.2 ± 3.1%) after 60 and 120 min. Research results showed that the caffeic acid contained in the capsules dissolved quickly but was limited to approximately 55%. No statistically significant difference (*p* > 0.05) was found between the solubility of caffeic acid gastric fluid and the intestinal fluid after 15 and 30 min. In view of the results obtained, it was hypothesized that the solubility of caffeic acid when administered orally would be better in gastric fluids compared to intestinal fluids. 

### 3.2. Solubility of Caffeic Acid 

In order to improve the solubility of caffeic acid, excipients were selected that are suitable for use in capsule manufacturing. The compositions of the mixtures of caffeic acid and excipients are given in Table 1. 

**Figure 2:**
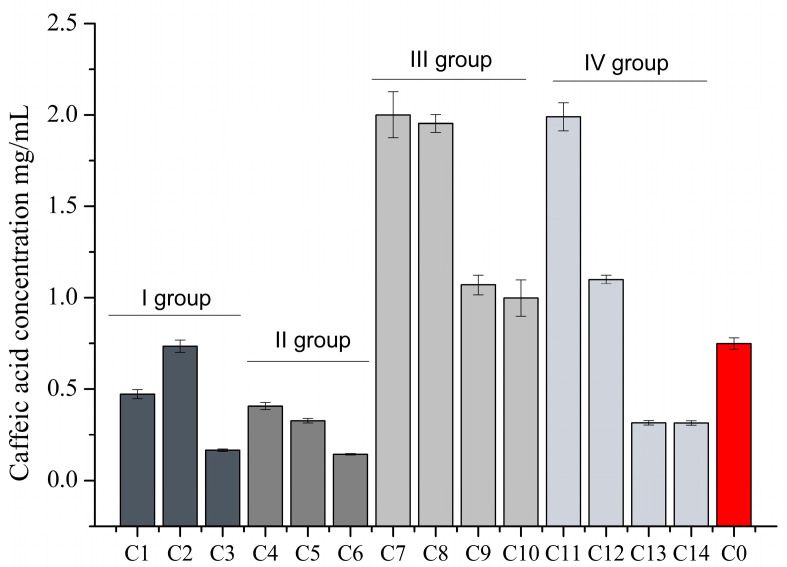
Solubility of caffeic acid into purified water solution containing different excipients and their mixtures (mean, SD, n = 3).

The effect of excipients on the solubility of caffeic acid in purified water was investigated. The results of the caffeic acid solubility study are shown in Figure 2.

The effect of the selected capsule fillers on the solubility of caffeic acid was found to be similar to that of group I fillers, which did not improve the solubility of caffeic acid (Figure 2. A higher amount of caffeic acid was found in the sample containing 100 mg of caffeic acid and Prosolv 50 mg (C2), which was not statistically significantly different from the control. The lowest dissolved caffeic acid content (1.64%) was found in the C3 sample containing 100 mg caffeic acid and 50 mg starch. The samples with hypromellose (group II) had the lowest dissolved caffeic acid compared to the other groups studied (Figure 2). It was found that the solubility of caffeic acid decreased with increasing amounts of hypromellose in the mixture. The statistically significant (*p* < 0.05) lower dissolution of caffeic acid was observed in samples C4, C5, and C6 compared to the control. When the solubility results of group III were evaluated, it was found that poloxamer improved the solubility of caffeic acid (Figure 2). The highest solubility of caffeic acid was found in sample C8 with 100 mg of caffeic acid and 50 mg of poloxamer in a ratio of 2:1. No statistically significant difference was found between the dissolved content of caffeic acid in samples C7 and C8. Increasing the amount of poloxamer in the samples at 1:1 and 1:1.5 decreased the solubility of caffeic acid. When assessing the solubility of caffeic acid in the samples of group IV, it was found that the addition of the solubility-enhancing agent β-cyclodextrin could improve the solubility of caffeic acid (Figure 2). Sample C11, containing 25 mg Prosolv and 25 mg β-cyclodextrin, showed the highest dissolution of caffeic acid with a solubility of 19.90%. The results showed that the solubility of caffeic acid decreased with increasing β-cyclodextrin content in the samples. 

### 3.3. Disintegration Test of Capsules Containing Caffeic Acid In Vitro

**Figure 3:**
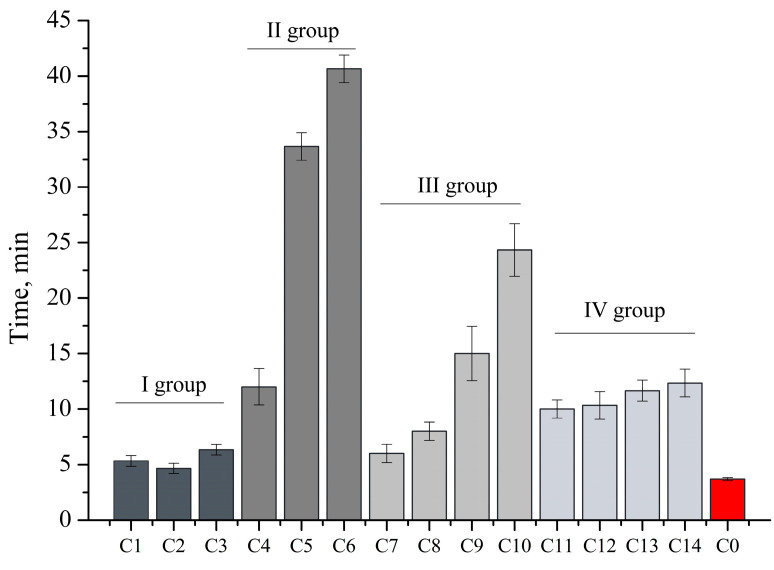
Disintegration time (min) of capsules containing caffeic acid (mean, SD, n = 3).

The disintegration test of capsules is one of the most important quality indicators for capsules and is regulated by the European Pharmacopoeia. The results of the capsule disintegration test are shown in Figure 3.

The results of the experimental study showed that the fastest degradation was observed for the capsules of groups I and IV. Group II capsules containing hypromellose had the longest disintegration time. The results showed that the disintegration time slowed down with increasing hypromellose content. It can be concluded that hypromellose prolongs the disintegration time of capsules. In terms of disintegration time, the capsules of groups I, III, and IV and the capsules of group II with C4 composition disintegrated in less than 30 min. Capsules C5 and C6 took longer than 30 min to disintegrate, which is attributed to the content of hypromellose and its ability to prolong action.

### 3.4. Dissolution Test of Capsules Containing Caffeic Acid In Vitro

**Figure 4:**
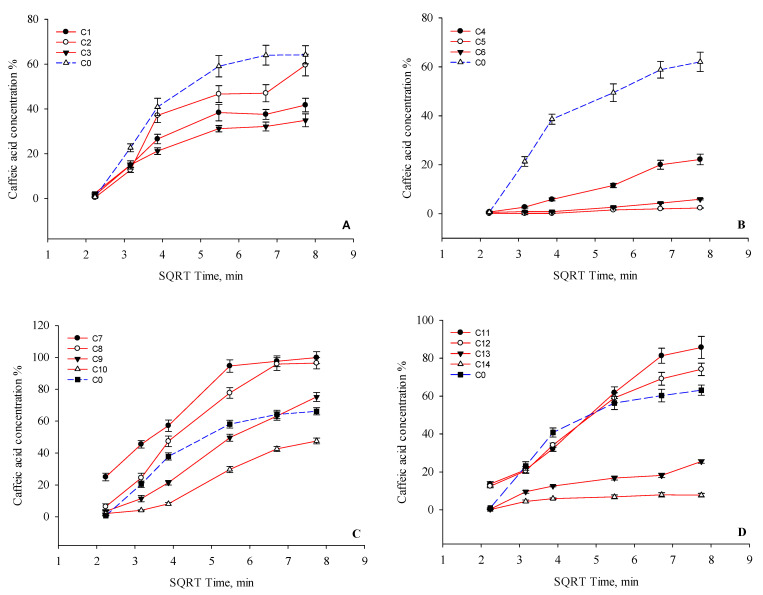
Results of the dissolution test. Percentage release of caffeic acid verses square root of time from the C0–C14 capsules after 60 min. (**A**)—I group, (**B**)—II group, (**C**)—III group, and (**D**)—IV group based on Table 1.

Dissolution test studies have shown that the capsules exhibit different dissolution kinetics of caffeic acid. The results of the test are shown in Figure 4.

The group I capsules dissolved a lower amount of caffeic acid compared to the control capsule C0 (Figure 4A). However, there was no statistically significant (*p* > 0.05) difference between the amount of caffeic acid released from capsules C0 and C2. The results show that the choice of filler had an influence on the dissolution of caffeic acid from the capsules produced. Capsules C2 released a statistically significantly (*p* < 0.05) higher amount of caffeic acid compared to capsules C1 and C3. A mathematical analysis of the kinetic profile of caffeic acid showed that the coefficients of Higuchi model regression for capsules C1, C2, and C3 were 0.8585, 0.8744, and 0.8826, respectively. The capsules of the second group showed lower amounts of caffeic acid after 1 h of testing compared to capsules C0 (Figure 4B). The results showed that the release of caffeic acid slowed down as the amount of hypromellose in the capsule increased. Statistically significantly (*p* < 0.05) lower amounts of caffeic acid were released from capsules C4, C5, and C6 compared to the control capsules C0 after 60 min. Hypromellose prolonged the dissolution kinetics of caffeic acid from the modeled capsules. The Higuchi regression coefficients of formulations C4, C5, and C6 were 0.9810, 0.9356, and 0.9467, respectively, and C0 was 0.8660. Figure 4C shows that the highest amounts of caffeic acid were released from capsules C7 and C8. More than 85.0% of the caffeic acid was released from capsules C7 and C8 after 30 min. Capsules C9 and C10 released 75.2% and 47.6% of the active substance, respectively, after one hour. The results of the study show that poloxamer improves the solubility of caffeic acid. From capsules C7, C8, and C9, a statistically significantly (*p* < 0.05) higher amount of caffeic acid was dissolved compared to capsules C0 and C10. The Higuchi regression coefficients of capsules containing poloxamers C7, C8, C9, and C10 were 0.9133, 0.852, 0.9919, and 0.9692, respectively. The results show that the amount of poloxamer has an influence on the dissolution kinetics of caffeic acid. From capsule C10, a statistically significantly (*p* < 0.05) lower amount of caffeic acid was dissolved compared to the other capsules in this group. The results showed (Figure 4D) that from capsules C11 and C12, a higher amount of caffeic acid was dissolved compared to the other capsules studied, whose regression coefficients of the Higuchi model were 0.9806 and 0.9714, respectively. This suggests that the amount of β-cyclodextrin in the capsule had an effect on the solubility of caffeic acid. The results showed that the mixture of Prosolv and β-cyclodextrin (C11) improved the solubility of the caffeic acid: after 30 min, more than 50% of the caffeic acid was released, and after 60 min—85%. From capsule C12, about 74% of the caffeic acid was dissolved after 60 min. A statistically significantly (*p* < 0.05) higher amount of caffeic acid was released from capsules C11 and C12 compared to capsule C0. The addition of 100 mg and 150 mg of β-cyclodextrin to the capsules was found to slow down the dissolution of caffeic acid (C13 and C14). The Higuchi model regression coefficients for formulations C13 and C14 were 0.9141 and 0.7550, respectively. From capsule C13, caffeic acid was released more intensively after 45 min. A statistically significant (*p* < 0.05) decrease in the amount of caffeic acid was observed in capsules C13 and C14 compared to capsules C0. Summarizing the results of the dissolution test for the capsules containing caffeic acid, it can be concluded that after 60 min, 80% or more of the caffeic acid dissolved from capsules C7, C8, and C11 containing this excipient, respectively: Prosolv 25 mg and poloxamer 25 mg; poloxamer 50 mg; Prosolv 25 mg and β-cyclodextrin 25 mg. Capsules C7, C8, and C11 met the requirements of the European Pharmacopoeia and showed a release of at least 80% of the active substance after 45 min.

## 4. Discussion

Given that most medicinal substances have poor solubility, researchers are looking for different solutions to this problem [42,43]. One of the most important factors is the ability of the solid form, administered orally, to dissolve in physiological body fluids and the therapeutic effect of the absorbed active substance [25,44]. Arivarasu et al. described in their study that orally administered caffeic acid is an effective agent in reducing the intestinal effects of Cisplatin and may be useful in reducing the gastrointestinal toxicity of this drug [45]. In the Dikmen et al. study, solid lipid nanoparticles enhanced the effects of caffeic acid on MCF-7 cells [46]. The results of our study confirmed that caffeic acid has limited solubility in water and in fluids mimicking physiological conditions [14]. The solubility of caffeic acid was improved by poloxamer 407 and β-cyclodextrin. The best solubility of caffeic acid was obtained when the amounts of these excipients in the samples were 25 mg and 50 mg. An analysis of the scientific literature has shown that both β-cyclodextrin and poloxamer have solubility-enhancing properties of the active ingredient and affect the dissolution kinetics of poorly soluble substances [8,13,32,33]. 

The in vitro digestive process typically involves the use of simulated gastric fluid (SGF) and simulated intestinal fluid (SIF), which simulate the conditions of the stomach and small intestine, respectively. A capsule is inserted into the SGF and allowed to simulate stomach digestion, during which the pH is gradually reduced and enzymes such as pepsin are added. The capsule is then transferred to the SIF, where simulated intestinal digestion takes place, during which the pH is gradually increased and enzymes such as pancreatin are added [41]. The results of in vitro digestive tract studies can provide valuable information on the release characteristics of capsules. For example, such studies can reveal whether capsule contents are released quickly or slowly, and whether factors such as pH and enzyme activity affect the release [41]. In this study conducted, the solubility of caffeic acid was investigated in simulated gastric and intestinal fluids using in vitro digestion of the digestive tract. The results showed that significantly more caffeic acid was dissolved in the gastric fluid (54.4 ± 2.1%) than in the intestinal fluid (45.2 ± 3.1%) after 60 and 120 min (*p* < 0.05). No statistically significant difference (*p* > 0.05) was found between the solubility of caffeic acid in the stomach fluid and digestion fluid after 15 and 30 min. These results suggest that the solubility of caffeic acid in the gastric fluid is better than in intestinal fluids when it is consumed orally, as more caffeic acid is dissolved in the stomach fluid during simulated digestion. However, it should be noted that this study was conducted in vitro, and the results may not necessarily reflect in vivo conditions. Further studies, including in vivo studies, are needed to confirm these conclusions. The results of the capsule weight uniformity test met the quality requirements of the European Pharmacopoeia monograph [47]. The weight deviation was within the 10% limits set by the European Pharmacopoeia. The results of the study show that the selected excipients ensure proper bulking and dosing of the powder mixture into capsules. In vitro disintegration and dissolution tests are important for the evaluation of the quality of the capsules and are described in the European Pharmacopoeia [47]. These tests have been applied to the quality assessment of simulated capsules containing caffeic acid. The evaluation of the disintegration time of the capsules produced showed that the capsules containing hypromellose 100 mg (C5) and 150 mg (C6) had a longer disintegration time than the European Pharmacopoeia required, i.e., they disintegrated in more than 30 min. The other capsules met the quality requirements. The dissolution test results showed that the dissolution kinetics of caffeic acid were influenced by the choice of excipients. Glucose is very soluble in water [48], but this did not have a positive effect on the dissolution kinetics of caffeic acid. The addition of starch also had an effect on the reduction of the solubility of caffeic acid. Given that starch is insoluble in cold water but forms a paste (a complex polysaccharide) in hot water, which increases the viscosity of the solution when dissolved [49,50], it may have an effect on the results of the solubility of caffeic acid. The results of the study confirmed that hypromellose prolongs the dissolution kinetics of caffeic acid. After one hour, group II capsules released the lowest amount of caffeic acid compared to the other capsules tested. In future research, it is important to solve the problem of the limited solubility of caffeic acid by modeling extended-release capsules. It is appropriate to extend this study by modeling capsules from which 20–30% of caffeic acid would dissolve within 60–120 min, and at the following points: 50% and at least 80%. Poloxamers are non-ionic polyoxyethylene-polyoxypropylene copolymers mainly used in solid formulations as emulsifiers or solvents [51,52]. The results of the research confirmed that the solution of poor solubility is the use of excipients that improve solubility. During this study, excipients were used to improve the solubility of caffeic acid and ensure the required dissolution kinetics. In order to improve the release kinetics of caffeic acid from the produced capsules, a solubility enhancer, poloxamer, was used. Poloxamers have an amphiphilic structure, hydrophilic properties, and the ability to self-aggregate to form micelles and a liquid crystal phase. The poorly water-soluble drugs are dissolved in the core of the formed micelle or conjugated to the micelle-forming polymer. The results of the study showed that the poloxamer improves the dissolution kinetics of caffeic acid, but the amount of poloxamer used is also influential: as the amount of poloxamer in the capsule increases, the dissolution of caffeic acid slows down. Poloxamers swell and gel in water, thus prolonging the release of the active substance from the solid form [33]. Given this property, it may have influenced the dissolution kinetics of caffeic acid from the capsules manufactured with higher levels (100 mg and 150 mg) of poloxamer (C9, C10). Another excipient used to improve the dissolution kinetics of caffeic acid was beta-cyclodextrin and its different levels in the simulated capsules. The results of the study showed that cyclodextrin improves the dissolution of caffeic acid at 50 mg and 100 mg in the capsule. The CD molecule contains a central cavity, the structure of which consists of hydrophobic functional groups located inside the cavity and hydrophilic functional groups located outside the molecule [30,51,53]. The positioning of the hydroxyl groups in the CD allows the CD to form complexes with the drug molecules, improving drug solubility and bioavailability [51,53]. These solubilizers are suitable components in the modeling of nanostructured carriers to ensure good solubility of caffeic acid. Caffeic acid has poor bioavailability, so nanostructured carriers are one of the future solutions to improve its penetration through biological membranes [54,55]. In order to more fully understand their effect on the solubility of caffeic acid, a dissolution kinetics test was performed. The results of this study suggest that it is important to assess the quality of the capsules by in vitro dissolution testing. This is a key analytical test to assess not only the dissolution kinetics of the drug substance, but also the influence of excipients on the release and solubility of the drug substance from the solid form [56]. The results of the studies show the importance of assessing the influence of excipients on the dissolution kinetics of the drug substance when selecting excipients for the manufacturing of capsules. This is particularly important when the drug substance has poor solubility. Of the fourteen capsule formulations produced, only two capsules (C7 and C8) released 80% or more of the caffeic acid content after 45 min. These capsules complied with the requirements of the European Pharmacopoeia. The other capsules (C9, C11, C12) showed a slower dissolution of the caffeic acid, with about 80% or more of the caffeic acid dissolved after 60 min. The results of the study substantiated the importance of dissolution testing in assessing the quality of the caffeic acid capsules produced. The results of the dissolution kinetics test are relevant when the active substances have poor solubility. When substances have poor solubility during this test, by replicating the physiological conditions of the body, it is possible to predict the solubility of the active compounds in the body. Based on the scientific data of the literature, when modeling the oral form with a material of poor solubility, it is necessary to seek to improve its solubility and thus not prevent the effectiveness.

## 5. Conclusions

Caffeic acid is a promising component in oral formulations. The problems of the poor solubility of caffeic acid can be solved by using solubility-improving auxiliary substances. The results of the studies on the solubility of caffeic acid in different mixtures showed that the use of excipients has an influence on the solubility of the active substance. Poloxamer 407 and β-cyclodextrin improve the aqueous solubility of caffeic acid and its dissolution kinetics from the capsules, but the effect of these agents is dependent on their content in the mixture. The results of in vitro dissolution kinetic studies are an important selection tool for further bioavailability studies of quality formulations with caffeic acid. The application of innovative nanostructured carriers in the future would enhance the solution to the solubility and bioavailability problems of caffeic acid.

## Figures and Tables

**Table 1:** Composition of capsules containing caffeic acid.

Group	Sample	PSMCC^TM50^, mg	P407, mg	HPMC, mg	β-C, mg	TCM, mg	MM, mg	Filling Quality
-	C0	-	-	-	-	100	103	Capsules filled completely
I	C1	-	-	-	-	150	152	
	C2	50	-	-	-	150	149	
	C3	-	-	-	-	150	151	
II	C4	-	-	50	-	150	152	
	C5	-	-	100	-	200	202	
	C6	-	-	150	-	250	251	
III	C7	25	25	-	-	150	151	
	C8	-	50	-	-	150	153	
	C9	-	100	-	-	200	202	
	C10	-	150	-	-	250	251	
IV	C11	25	-	-	25	150	150	
	C12	-	-	-	50	150	149	
	C13	-	-	-	100	200	203	
	C14	-	-	-	150	250	248	

All modeled capsules contained 100 mg of caffeic acid; capsule C2 contained 50 mg of glucose; capsule C3 contained 50 mg of starch. CA—caffeic acid; PSMCC^TM50^—PROSOLV SMCC^TM50^; HPMC—hypromellose; P407—Poloxamer 407; β-C—β-cyclodextrin; CC—composition of capsules; TCM—theoretical mass of capsule; MM—mean mass of capsule.

## Data Availability

All the other data are available on reasonable request from the corresponding authors.
